# Biofunctionalized Nanofibers Using *Arthrospira* (*Spirulina*) Biomass and Biopolymer

**DOI:** 10.1155/2015/967814

**Published:** 2015-01-15

**Authors:** Michele Greque de Morais, Christopher Stillings, Roland Dersch, Markus Rudisile, Patrícia Pranke, Jorge Alberto Vieira Costa, Joachim Wendorff

**Affiliations:** ^1^Laboratory of Microbiology and Biochemical, College of Chemistry and Food Engineering, Federal University of Rio Grande, P.O. Box 474, 96203-900 Rio Grande, RS, Brazil; ^2^Department of Chemistry, Philipps-Universität Marburg, Hans Meerwein Straße, 35032 Marburg, Germany; ^3^Hematology and Stem Cell Laboratory, Faculty of Pharmacy, Federal University of Rio Grande do Sul, 90610-000 Porto Alegre, RS, Brazil; ^4^Laboratory of Biochemical Engineering, College of Chemistry and Food Engineering, Federal University of Rio Grande, P.O. Box 474, 96203-900 Rio Grande, RS, Brazil

## Abstract

Electrospun nanofibers composed of polymers have been extensively researched because of their scientific and technical applications. Commercially available polyhydroxybutyrate (PHB) and polyhydroxybutyrate-co-valerate (PHB-HV) copolymers are good choices for such nanofibers. We used a highly integrated method, by adjusting the properties of the spinning solutions, where the cyanophyte *Arthrospira* (formally *Spirulina*) was the single source for nanofiber biofunctionalization. We investigated nanofibers using PHB extracted from *Spirulina* and the bacteria *Cupriavidus necator* and compared the nanofibers to those made from commercially available PHB and PHB-HV. Our study assessed nanofiber formation and their selected thermal, mechanical, and optical properties. We found that nanofibers produced from *Spirulina* PHB and biofunctionalized with *Spirulina* biomass exhibited properties which were equal to or better than nanofibers made with commercially available PHB or PHB-HV. Our methodology is highly promising for nanofiber production and biofunctionalization and can be used in many industrial and life science applications.

## 1. Introduction

In recent years several innovative nanofiber technologies are being studied, such as the development of structural nanocomposites providing dual drug release through a combination of electrospinning and electrospraying [1] and the use of a modified coaxial electrospinning process in the production of drug loaded cellulose acetate nanofibers [2]. The use of nanofibers has also been associated with antibacterial functionality of graphene for applications in wound healing [3] and the development of methods for carbon nanotubes and carbon nanobelts via double needle electrospinning on a basis of water-in-oil emulsion technique [4].

Polymer nanofibers produced by electrospinning are currently receiving a great deal of interest, largely because of their technical and life science applications. These highly porous, nonwoven fibers have a large surface area and can be used for diverse applications, including the tissue engineering [5–10]. For effective tissue reconstruction, scaffolds must conform to specific requirements. High porosity and pore interconnectivity are fundamental characteristics for increasing the available specific surface area, which is important not only for cell anchorage and the internal growth of tissues but also for facilitating the distribution and transport of oxygen, nutrients, and cellular residues [11].

The degradability is a parameter closely related to the solubility of the molds. The degradability is associated with the stability of the biomaterial* in vivo* and an appropriate time is extremely important for proper regeneration [12]. The most appropriate scaffold material should be biocompatible and biodegradable, so that it is nonimmunogenic and to avoid further surgical intervention when tissue regeneration is complete [13].

Nanofibers can also be biofunctionalized; that is they can be altered for specific functions and thus improved, by, for example, incorporating compounds such as antioxidants, anti-inflammatory, antibacterial, antifungal, vitamins, or other compounds [14]. However, in addition to incorporating particular biofunctions into the nanofibers the properties, such as electrical conductivity, of the solutions used in spinning such fibers must be modified to obtain smaller diameter nanofibers which are homogeneous and free from beads and have better permeation characteristics.

Polyhydroxybutyrate (PHB) and its copolymer with hydroxyvalerate (HV), poly(3-hydroxybutyrate-co-3-hydroxyvalerate) (PHB-HV), are accumulated by some microorganisms where they act as intracellular storage compounds which can be used as a source of carbon and energy [15, 16]. Commercially available PHB is a thermoplastic polyester which is nontoxic, biodegradable, and biocompatible with tissues, because of which it has specialized applications in areas such as medical technology [17]. In mammalian tissues, degradation products are absorbed through the cellular wall and metabolized [18]. The degradation rate of PHAs depends on many factors. Some factors, such as temperature, humidity, pH, and nutrient supply, are related to the environment, while other factors, such as additives composition, crystallinity, and surface area, are intrinsic to the biopolymer [19].

Microalgae and cyanophytes are photosynthetic microorganisms that can double their biomass in 24 hours and not only produce PHB polymers but also biologically active compounds such as proteins, polyunsaturated fatty acids, vitamins, pigments, antioxidants. Cyanophytes from the genus* Arthrospira*, usually* Arthrospira platensis* and* Arthrospira maxima*, have for many years been commercially available under the name “*Spirulina*”, which has been reported to be not only therapeutically effective in reducing cholesterol levels but also displaying antimutagenic and antiviral activity, potentially inhibiting HIV replication [20] and tumor cells [21].

We used PHB extracted from different types of microorganisms to develop nanofibers and used electrospinning to produce uniform nanofibers, which were then biofunctionalized by the incorporation of* Spirulina* biomass. Commercially available PHB-systems were also studied for comparison.

## 2. Materials and Methods

Nanofibers were prepared from PHB extracted from* Spirulina platensis* (kindly provided by Dr. Jau, School of Biological Sciences, Universiti Sains Malaysia, Pulau Pinang, Malaysia) and* Cupriavidus necator* bacterial PHB samples RE and CN (kindly provided by G. Aragão, Universidade Federal de Santa Catarina, Santa Catarina, Brazil); commercially available PHB (Sigma-Aldrich, USA), and PHB containing poly(3-hydroxyvalerate 5% (w/w) (PHB-HV5, 1.93 × 10^6^ Da) and 12% (w/v) (Sigma-Aldrich, USA) were also used for comparison. Nanofibers were electrospun from chloroform solutions of PHB and PHB-HV, with the properties of the spinning solutions and the resulting nanofibers being modified by adding from 0.2% to 2.2% (w/w) sodium chloride and 5% to 35% (w/w)* Spirulina* biomass (Table 1). The samples were homogenized in a model MR 3001 K magnetic stirrer (Heidolph Instruments GmbH & Co., Schwabach, Germany) at 300 rpm for 12 hours at 21.0°C.

Biofunctionalization (Table 1) was accomplished using* Arthrospira* biomass [22] cultivated in a pilot plant as previously described [23]. The* Spirulina* biomass was harvested using a 200 *μ*m diameter filter and then concentrated and extruded using a hydraulic press. After extrusion, the biomass was dried at 50°C for 4 h in a tray dryer ground in a model Q-298-2 ball grinder (Quimis, Brazil), vacuum packed using a model Supervac400 vacuum packing machine (Suplack, Brazil), and stored in the dark at 4°C. The amount of ash, lipid, moisture, and protein in the biomass was measured using standard methods [24].

Electrospinning was carried out at 21°C by injecting the PHB solutions (Table 1) through 0.45 mm or 0.60 mm diameter capillaries at a flow rate of 0.7 *μ*L min^−1^ to 6.5 *μ*L min^−1^. The capillaries had a positive electrode at their tip and a grounded aluminum collector between 150 mm and 200 mm from the tip and an applied electric potential ranging from 12.4 kV to 31.3 kV. To ascertain the best conditions for producing uniform nanofibers, the PHB and PHB-HV samples were tested at different concentrations, voltages, flow rates, distances from the capillary to the collector, and capillary diameters (data not shown).

The apparent viscosity of the spinning solutions was determined using a model PK100 viscometer (Haake, Germany) and the conductivity of the solutions with a digital conductivimeter (Inolab, Germany), respectively. Nanofiber mean diameter was calculated using a JSM-7500F scanning electron microscope (Jeol, Germany) to measure 30 different points across SEM images of the nanofibers and mean nanofiber diameters being subjected to analysis of variance (ANOVA) and the Tukey test (*P* ≤ 0.05). The mechanical properties of the nanofibers were evaluated using sets of parallelly orientated nanofibers spun on an electrode rotating. Stress-strain experiments were done by assessing elasticity, tensile strength, and breaking elongation using a BT1-FR0.5TN.D14 mechanical analyzer (Zwick, Germany), with mean values being subjected to ANOVA and the Tukey test (*P* ≤ 0.05). Molecular weight of the polymers was determined by gel permeation chromatography (GPC) on a model SL1000 HPLC (Knauer, Germany) equipped with a Polymer Standard Services column and two Knauer detectors (K2500 UV for detector 1 and RI for detector 2) and using hexafluoroisopropanol (Aldrich, Germany) as the mobile phase solvent at a flow rate of 0.5 mL min^−1^ and 23°C with a 100 *μ*L sample injection volume, with molecular weight standard being 319,000 Da polystyrene (Aldrich, Germany). The thermal degradation and degree of impurity of the polymers were measured using a model SDTA851 thermogravimetric analyzer (TGA) (Mettler, Germany) and approximately 10 mg of sample heated from 25°C to 700°C at 10°C min^−1^ in air with nitrogen, with the initial and highest degradation temperatures being determined from the first derivative of the TGA curves and the degree of impurities being characterized from the amount of sample remaining as ash at the end of the process [25]. The degree of crystallinity (*χ*_*c*_) of the polymers was calculated from the enthalpy of crystallization (Δ*H*_*c*_) and the enthalpy of melting (Δ*H*_*m*_) by differential scanning calorimetry (DSC) using a model DSC821e calorimeter (Mettler, Germany) and 10 mg polymer samples sealed in aluminum capsules and heated and cooled between 25°C and 200°C at a rate of 10°C min^−1^, with the enthalpy of melting 100% crystalline PHB and PHB-HV (142 J g^−1^) being used as a standard [26].

## 3. Results and Discussion

The electrospinning process and resultant nanofiber formation are known to depend strongly on the properties of the solutions from which they are spun, such as electric conductivity and viscosity. All the solutions prepared without sodium chloride or added* Spirulina* biomass showed conductivities ranging from 1 × 10^−4^ mS cm^−1^ to 1 × 10^−6^ mS cm^−1^, whereas solutions modified by the addition of NaCl or* Spirulina* biomass had conductivities that were higher by many orders of magnitude, up to 1 mS cm^−1^, while the viscosities of the different spinning solutions showed little variation irrespective of the presence or absence of sodium chloride or* Spirulina* biomass (Table 2).

**Figure 1:**
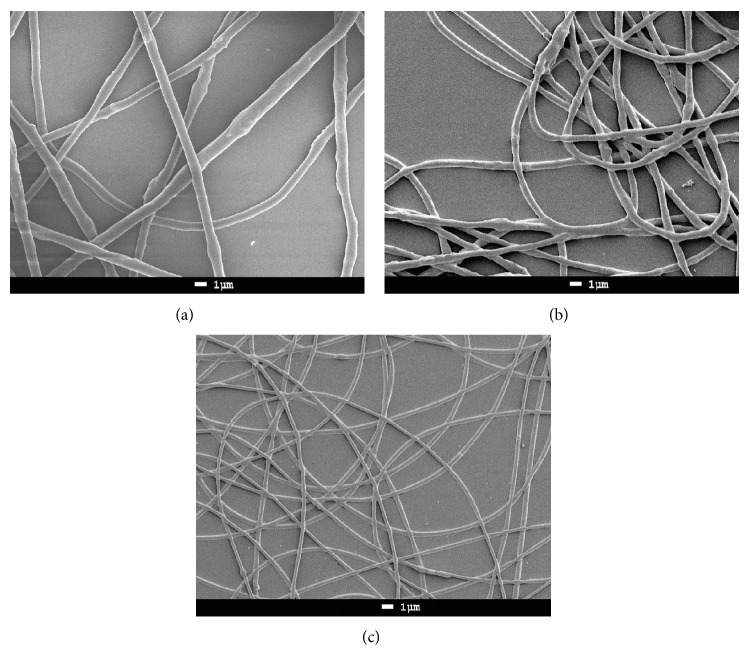
Scanning electron microscopy of nanofibers produced with solutions containing (a) 22% w/w PHB extracted from* Spirulina platensis* (SP1), (b) 22%* Spirulina platensis* PHB and 2.2% sodium chloride (SP2), and (c) 7%* Spirulina platensis* PHB and 5%* Spirulina* biomass (SP3). 4,000x magnification.

The PHB extracted from* Spirulina* and commercial PHB-HV5 and PHB-HV12 were electrospun with and without the addition of sodium chloride or* Spirulina* biomass (Table 1). Electrospinning of 22% w/w* Spirulina* PHB without the addition of sodium chloride or* Spirulina* biomass produced uniform nanofibers with a diameter of about 750 nm, while the addition of sodium chloride reduced the nanofiber diameter to about 480 nm and the addition of 5% w/w* Spirulina* biomass reduced it to about 310 nm (Table 1, Figure 1). It is important to note that if biomass is added to the spinning solution, PHB nanofibers can be spun with PHB concentrations as low as 7% w/w. This produces nanofibers with markedly reduced fiber diameters, which could be of importance in regard to the properties of membranes produced from such nanofibers. One reason for the reduced nanofiber diameter in the presence of* Spirulina* biomass may have been that the biomass also contained some PHB, although this cannot be the main reason because the amount of biomass was small. This phenomenon needs further research. Spinning nanofibers using lower concentrations of PHB would reduce production costs.

To obtain the best conditions for producing uniform nanofibers we tested the PHB and PHB-HV samples using several PHB concentrations and different capillary diameters, flow rates, voltages, and capillary to collect distances. The commercial PHB sample (sample B) and the* C. necator* PHB sample CN did not form nanofibers when using solutions with PHB concentrations of about 20% w/w, while the* C. necator* PHB sample RE produced nanofibers with droplets, because of which these samples were not tested with sodium chloride and* Spirulina* biomass. The 20% w/w PHB-HV5 and PHB-HV12 solutions produced larger diameter nanofibers of about 1200 nm (Table 1). It has been reported that PHB-HV solutions containing less than 13% PHB-HV formed nanofibers with droplets, while solutions containing 20% w/w PHB-HV produced nanofibers with a uniform diameter of between 1,000 nm and 4,000 nm [16]. We found that the addition of sodium chloride and/or biomass did not significantly reduce the diameter of nanofibers produced from PHB-HV5, despite a large increase in the conductivity of the spinning solutions and the reduced PHB-HV5 concentration which was possible due to the addition of salt (Table 1). However, the diameter of nanofibers spun from PHB-HV12 decreased significantly in size to about 1000 nm when sodium chloride was added and to about 800 nm when* Spirulina* biomass was added. The commercially available PHB did not produce nanofibers with diameters as small as those observed for PHB extracted from* Spirulina*.

Regarding thermal properties, the PHB-HV12 nanofiber sample had the lowest melting temperature, while Spirulina platensis PHB had lower initial and final decomposition temperatures than that seen for commercial PHB (Table 2). Final degradation temperatures of 263.5°C for PHB and 265.5°C for PHB-HV have been reported [25]. Thermal degradation analysis of PHB and PHB-HV can characterize the level of impurities produced during the cultivation of the microorganism and the extraction of the PHB, such impurities affecting the color, smell, and sheen of the final product [27]. The maximum and minimum melting temperatures of the PHB and PHB-HV samples are shown in Table 2. Similar values of 172.6°C for PHB and 158.7°C for PHB-HV have been reported [25], while the melting temperature of PHB nanofibers has been reported as 165°C [28].

Regarding impurities, the PHB produced from* Spirulina* and the commercial samples B showed the lowest levels of impurity at 1.3%, while* C. necator* sample RE showed the highest at about 4% w/w (Table 2). The final* Spirulina* biomass consisted of 86% w/w protein, 6.7% ash, 5.3% moisture, and 0.2% lipids (Table 2). The molecular weight of the PHB and PHB-HV samples is shown in Table 2. The molecular weight of PHB is known to vary according to the type of microorganism and the different stages and conditions of cultivation, varying between 1.0 × 10^4^ and 3.0 × 10^6^ Da for bacterial PHB [29].

The degree of crystallinity of the PHB and PHB-HV nanofiber samples in our study is given in Table 2, from which it can be seen that the PHB-HV12 sample had the lowest degree of crystallinity. Poly(3-hydroxybutyrate) is a rigid, brittle, material with a degree of crystallinity ranging from 60% to 80%, while the degree of crystallinity for PHB-HV varies between 50% and 70.0% [30]. These values have been confirmed by other authors, who reported that the degree of crystallinity was 53.1% for PHB and 51.8% for PHB-HV [25].

The mechanical properties (elasticity, tensile strength, and breaking elongation) for the PHB and the copolymer PHB-HV nanofiber samples used in our study are shown in Table 3. The elasticity of* Spirulina platensis* PHB nanofibers showed the highest value at about 253 Mpa, while the elasticity of that of the copolymer samples was about half this value (Table 3). The addition of* Spirulina* biomass strongly reduced the elasticity in all cases, and there was a particularly large drop for* Spirulina* PHB (Table 3). Nanofibers made from commercial PHB have been reported as having an elasticity of 147.3 Mpa [31]. Tensile strength was particularly high for* Spirulina* PHB and there was a marked reduction when* Spirulina* biomass was present (Table 3). Nanofibers made from commercial PHB have a reported tensile strength of 1.8 MPa [25]. The breaking elongation value for nanofibers made from* Spirulina* PHB was 7%, almost three times that of the commercial PHB and PHB-HV samples (Table 3). Addition of* Spirulina* biomass strongly reduced the breaking elongation for* Spirulina* PHB samples while not significantly altering the values for the other samples. Nanofibers made from commercial PHB have been reported as having a breaking elongation of 2.3% [31]. The general finding is that nanofibers composed of* Spirulina* PHB possess surprisingly enhanced mechanical properties as compared with nanofibers composed of commercial PHB. These properties become markedly reduced by the addition of biomass.

**Figure 2:**
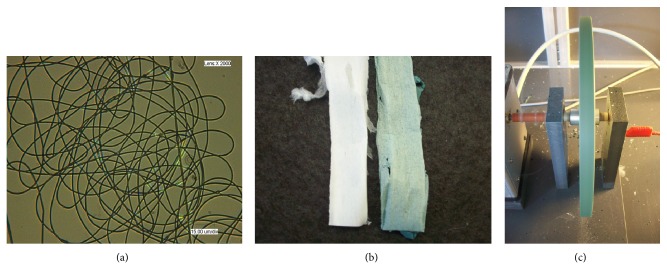
Optical image of PHB nanofibers containing* Spirulina* biomass incorporated, 2,000x magnification (a), nanofibers of PHB without and with* Spirulina* biomass incorporated developed in rotatory collector (b), and PHB nanofibers containing* Spirulina* biomass incorporated before removing the rotatory collector (c).

The optical properties of nanofibers are important in applications such as protection from injury, where blocking light leads to a loss of pigments, antioxidants, and other components like the scaffolds. However, the addition of* Spirulina* biomass can provide additional optical functionalization of the nanofibers and affect the transmission of light because addition of biomass produces a nanofiber with a strong green color (Figure 2).

## 4. Conclusions

Some highly innovative life science applications that have been recently developed, such as tissue engineering, have created a demand for nanofibers composed of biocompatible, biodegradable polymers on a technical scale. The study evaluated nanofiber formation, thermal and mechanical properties, and selected optical properties and compared them with PHB systems from other sources. The conclusion is that the integrated approach taken here is highly promising in terms of nanofiber production, biofunctionalization, and life science applications. Nanofibers produced from PHB extracted from* Spirulina* had the smallest diameter (about 300.0 nm) in samples with added microalgal biomass, as well as enhanced elasticity of about 250.0 MPa, a tensile strength of about (8.0 MPa), a breaking elongation of about 7.0%, and minor impurities (1.3%). The reason for the deterioration of the mechanical properties of the nanofibers biofunctionalized with* Spirulina* biomass was probably due to biomass aggregation resulting in a heterogeneous distribution of biomass in the fibers. The properties of the nanofibers made from Spirulina PHB were highly favorable when compared to nanofibers produced from bacterial and commercial PHB and PBH-HV. The methodology described in this paper can be adapted to develop nanofibers that can be used in tissue engineering. Further studies should aim at reducing nanofiber diameter even more so that it approaches the ideal Knudsen number of kn ≥ 1 and to enhance the mechanical properties of biomass-containing nanofibers.

## Figures and Tables

**Table 1:** Source of polyhydroxybutyrate (PHB) and polyhydroxybutyrate-co-valerate (PHB-HV) and the composition of the solutions used to manufacture nanofibers constructed from PHB or PHB containing 5% or 12% (w/w) commercially available PHB-HV and biofunctionalized using *Arthrospira* biomass. Nanofiber diameters are given as the mean ± standard deviation.

PHB source and sample code	Concentration of PHB and *Spirulina* biomass (%, w/w)	Nanofiber type and diameter (mm)
*Spirulina platensis *		
SP1	22% PHB	Uniform, 744 ± 99
SP2	22% PHB + 2.2% NaCl	Uniform, 474 ± 80
SP3	7% PHB + 5% biomass	Uniform, 312 ± 68
Commercial		
B	20% PHB	No nanofibers produced
5V1	20% PHB-HV5	Uniform, 1205 ± 392
5V2	20% PHB-HV5 + 0.2% NaCl	Uniform, 1184 ± 225
5V3	15% PHB-HV5 + 5% biomass	Uniform, 1107 ± 279
12V1	20% PHB-HV12	Uniform, 1249 ± 169
12V2	20% PHB-HV12 + 0.2% NaCl	Uniform, 975 ± 177
12V3	15% PHB-HV12 + 5% biomass	Uniform, 835 ± 244
*Cupriavidus necator *		
CN	20% PHB	No nanofibers produced
RE	40% PHB	No droplets

**Table 2:** Source of polyhydroxybutyrate (PHB) and polyhydroxybutyrate-co-valerate (PHB-HV) and the physical characteristics of nanofibers constructed from PHB or PHB containing 5% or 12% (w/w) commercially available PHB-HV and biofunctionalized using *Spirulina* biomass.

PHB source, sample code, and composition	Conductivity (mS cm^−1^)	Viscosity (*η*, Pa s)	Melting temperature (*T*_*m*_, °C)	Initial degradation temperature (TG_0_, °C)	Final degradation temperature (TG_*f*_, °C)	Impurities (%, w/w)	Molecular weight (Da)	Crystallinity (*χ*_*c*_, %)
*Spirulina platensis *								
SP1, 22% PHB	1.1 × 10^−4^	1.1						
SP2, 22% PHB + 2.2% NaCl	7.7	1	175.8	232.4	329	1.3	1.5 × 10^6^	50.2
SP3, 7% PHB + 5% biomass	5.8	0.5						
Commercial								
B, 20% PHB	1.4 × 10^−4^	0.2	180	265.9	313.2	1.3	9.9 × 10^5^	57.2
5V1, 20% PHB-HV5	1.2 × 10^−4^	1						
5V2, 20% PHB-HV5 + 0.2% NaCl	6.3	0.9	176.7	243.7	279.9	3	1.9 × 10^6^	50
5V3, 15% PHB-HV5 + 5% biomass	4.7	0.7						
12V1, 20% PHB-HV12	1.5 × 10^−4^	0.65						
12V2, 20% PHB-HV12 + 0.2% NaCl	7	0.6	149.5	252.5	287.2	2	9.2 × 10^5^	21.3
12V3, 15% PHB-HV12 + 5% biomass	5.9	0.4						
*Cupriavidus necator *								
CN, 20% PHB	9 × 10^−6^	0.3	157.3	276.7	329	1.7	2.4 × 10^6^	40.8
RE, 40% PHB	2 × 10^−6^	0.5	180.1	238.5	279.7	4.1	5.8 × 10^5^	74.7

**Table 3:** Polyhydroxybutyrate (PHB) and polyhydroxybutyrate-co-valerate (PHB-HV) nanofiber elasticity, tensile strength, and breaking elongation. Means ± standard deviation.

PHB source and sample code	Elasticity (*E*_mod⁡_, Mpa)	Tensile strength (*σ*_*b*_, Mpa)	Breaking elongation (*E*_*b*_, Mpa)
*Spirulina platensis *			
SP1, 22% PHB	253 ± 34.2	8.1 ± 0.9	7.2 ± 1.2
SP3, 7% PHB + 5% biomass	40.1 ± 6	0.8 ± 0.2	0.8 ± 0.0
Commercial			
5V1, 20% PHB-HV12	116.3 ± 0.6	6 ± 0.8	2.8 ± 0.7
5V3, 15% PHB-HV5 + 5% biomass	56.7 ± 5.6	3.2 ± 0.0	2.6 ± 0.6
12V1, 20% PHB-HV12	123.7 ± 37.6	3.3 ± 0.9	1.7 ± 0.4
12V3, 15% PHB-HV12 + 5% biomass	97.9 ± 25.3	3.7 ± 0.9	2.6 ± 1.3
